# Institutional Notes and News

**Published:** 1909-11-20

**Authors:** 


### Institutional Notes and News.

### EARL CADOGAN AT BURY.

The new balconies presented to the West Suffolk General Hospital, Bury St. Edmunds, by Mr. and Mrs. Washington Charters, and the out-patients' department were opened for use on October 23 by Earl Cadogan, the hospital's president. The out-patients' department has cost £550, of which all but £20 has been already subscribed. The balconies are 78 feet long by 10 feet 6 inches, with reinforced concrete floors covered by red encaustic tiles.

### THE SALFORD BOYCOTT.

The medical boycott at the Salford Poor Law Infirmary, at Hope, is likely to be shortly removed, as the Guardians, after conferring with Mr. A. B. Lowry, the Local Government Board inspector for the district, and Dr. Fuller, medical inspector of the Local Government Board, have agreed to the appointment of a medical superintendent with sole administrative charge. A committee to consider the medical staffing of the Infirmary has been appointed.

### OLDHAM INFIRMARY.

It was recently decided to open a subscription list in order to purchase a portrait of the late Mr. John Dronsfield to be hung in the Oldham Art Gallery. The appeal has been so generously responded to that more money has being received than will be required for the purpose, and it has now been decided to try and raise £500 to endow a cot in the Infirmary to the memory of Mr. Dronsfield. Over £200 has already been received.

### NATIONAL HOSPITAL, QUEEN SQUARE.

The Secretary of the National Hospital for the Paralysed and Epileptic has received a letter, dated November 4, from Lord Knollys, who says :—"I think you would like to know that the King was much pleased with the arrangements connected with the ceremony at the hospital to-day. His Majesy thought that everything went off as well as possible." On Thursday an unknown friend of the hospital made a donation of £5,000 to the Jubilee Fund, bringing the total received to date to £25,000. He also gave £2,200 for general purposes.

### HADDON HOSPITAL.

The Haddon Hospital Committee has persistently declined to conform to the County Council's requirements in respect of the erection of a large central isolation hospital, in view of the preference of the local authorities concerned for a scheme of cottage hospitals. In June last the County Council sent an ultimatum threatening the Haddon Committee with extinction failing compliance within a week. On the advice of the Clerk to the Baslow and Bakewell Urban Councils (two of the constituent authorities) the Haddon Committee repudiated the County Council's claim to paramount powers, and a month ago the county medical officer sought in vain to pacify this defiant committee, or, at any rate, to effect a compromise. Now, in a letter couched in conciliatory terms strangely contrasting with the dictatorial mandate of four months ago, the Clerk to the County Council frankly recognises the Haddon Committee's independence and freedom to carry out its duties in its own way. As the members of the Haddon Committee readily recognised, remarks the *Sheffield Telegraph*, there is so striking a divergence in the attitudes assumed by the County Council in June and October as to justify the demand for a formal withdrawal from the position taken, and now apparently recognised by the County Council to be an impossible one.

### LEEDS MATERNITY HOSPITAL.

The quarterly meeting of the Leeds Maternity Hospital was held last week, the Lady Mayoress (Mrs. F. J. Kitson) presiding. The hon. treasurer's report showed a balance at the bank, and the total receipts amounted to £1,061, expenses to date amounted to £661, leaving a balance in hand of £400. The amount paid or promised to the Building Fund of the new hospital was £3,464, of which £2,160 has been paid out to builders and contractors, leaving a balance of £1,082. The house in Hyde Terrace which is being converted into the new hospital was given by Mr. Pepper, but new wings have had to be added, and the total cost of the alterations and furnishing is estimated to be about £10,000. There thus remains a deficit of between £6,000 and £7,000, and the question of the best means of raising this amount provided a lengthy discussion. Various suggestions were put forward, and eventually it was decided to form a committee in order that they might decide on the most suitable way of raising the money. The opening ceremony will take place next May.

### ROYAL NATIONAL ORTHOPÆDIC HOSPITAL.

The annual Sale of Work organised by the Ladies' Committee will be held on the fourth floor of the new buildings of the Royal National Orthopædic Hospital on November 24 and 25 at 2 o'clock. Lady Lansdowne has consented to open it on the first day. The hospital will be open for inspection on both days. At present only the first floor, containing 45 patients, is open, but during this month the second and third floors are to be opened, to contain 130 patients; this will leave only the fourth floor empty, and it is hoped that before many months the funds will be forthcoming to open this also and so provide accommodation for 200 in-patients. There are more than 300 patients on the books of the hospital waiting admission, many of whom have already waited more than a year for a vacancy. There is no limit to the length of stay of a patient in this hospital, and the children remain in as long as the surgeons consider it desirable for the most effective treatment of the cases; as a result, marvellous cures are often effected which would not be possible in a general hospital where a bed cannot long be occupied by the same case. A special feature of the new buildings is the branch of the out-patient department, which has been fitted up as an exercise room and gymnasium for the patients, in and out, suffering from spinal curvatures, paralysis, stiff joints, etc. The gymnasium includes 14 Zander instruments, as well as the Swedish gymnastic appliances. Patients attend two or three times a week, as may be necessary, and after their exercises are frequently ordered shower baths or massage or both, and in some cases hot-air baths and electrical treatment. The Zander apparatus was presented to the hospital by Mr. Otto Beit two years ago; during the erection of the new buildings it was fixed in an old tailor's shop in the temporary buildings, but the space here was very limited, and it was difficult for many of the patients to go up and down stairs; the new department is large and well ventilated, and is conveniently situated on the ground floor. Early in the New Year a school of massage and medical gymnasium is to be organised. The Royal National is now the only orthopædic hospital in London and the most up to date in the kingdom in its arrangements for the treatment of all deformities.

[We hope to publish a full account, with plans, of this hospital in an early issue.]

